# Alcoholic Beverages

**Published:** 1912-12

**Authors:** 


### Alcoholic Beverages

This subject has been brought to our attention, particularly with reference to the appearance of advertisements of alcoholic beverages in the columns of this and other medical journals and in such a way as not only to excuse but to demand a statement of personal views. We may say, therefore that, not only personally, but by heredity, we stand not merely for temperance but for total abstinence and that this stand dates back about 120 years.

But, we believe that total abstinence from alcoholic beverages should have nothing to do with the question of the medicinal use of alcohol. It would be out of place to present here, a paper detailing our own notions with regard to the therapeutics of alcohol although, to prevent misunderstanding, it may be said that such a paper would probably represent the average views of the profession at large.

What we do want to emphasize is that opinions regarding the use of alcohol as a routine, should have nothing whatever to do with its prescription as a drug or therapeutic adjuvant, that the person under the influence of alcohol should have exactly the same social and legal status as one under the influence of morphine, or any other drug, and that the best interests of genuine temperance are served by putting the marketing of alcoholic preparations on exactly the same basis as the marketing of digitalis, cannabis indica, diphtheria antitoxin or any other medicament.

---

We should be very glad of assistance in reviewing numerous exchanges, in English, French, German, Swedish, Italian, Greek and Japanese. This work is good training, both in medicine and in language. We shall be pleased to hear from volunteers, who need not necessarily live in Buffalo.

We are very grateful for numerous expressions of approval. It should be understood that the value of a journal depends mainly on the cooperation of its readers and contributors. It is, therefore, not immodest to say frankly that the one thing necessary to further improvement is money. Bills were inclosed with the August issue and while individual subscriptions are very minor matters, in the aggregate, they are far more important than any one advertisement or group of advertisements. Prompt payment facilitates office work and leaves more time for bettering the reading matter. It is also required by the Post Office, of all journals enjoying the privilege of second-class rates and the allowance of sample copies depends on the paid-up subscriptions. In the long run, however, the strength of any periodical is its advertising—unless it has an "Angel" which is not the case with an independent journal. If our readers will pay attention to the advertisements, they will have a journal of the highest grade, in the news and scientific departments. Some advertisers are content with indirect evidence as to the value of publicity. Others want actual personal inquiries and orders. In any correspondence, it is fair to mention the periodical to which the publicity is due. It is best to do so by name as many advertisements—though only a few in this journal—are keyed. For example one business man told us that he deliberately gave a wrong address in a certain journal (not this one). Another does not understand most grown men know that catalogue K, S, W, etc., is all the same. Firms in large cities usually give only the city address, some not even this as they depend solely on wholesaling while firms in small towns often give explicit street addresses to key their advertisements. Others give the name of a building in one journal, a street number in another, a manager's name in another. Obviously, most busy men pay no attention to these details, their sole object is to get a letter to the place desired. The result is that, unless the name of the periodical is definitely mentioned, credit for publicity is lost. The reason why busy men are bothered with circular letters is not that they are cheaper than legitimate advertising; not that they produce good results (one firm reported to us that they received inquiries—not orders—from only one per cent. of circular letters); but that the results can be accurately checked.

---

Apology for Errors. Quite a number of errors in typography occurred in the November issue. These were corrected on proof but, in some way, the revised proof was overlooked. Considering the fact that there was an almost complete change of personnel at the printing office and that the journal was unfamiliar to all, in addition to the fact that a rush of other work made it necessary to hurry over details, it is surprising that more mistakes were not made.

